# Relationship Between Ocular Trauma Score and Computed Tomography Findings in Eyes with Penetrating Globe Injuries: A Preliminary Study

**DOI:** 10.3390/diagnostics15070830

**Published:** 2025-03-25

**Authors:** Berire Şeyma Durmuş Ece, Zübeyir Yozgat, Yusuf İnançlı, Bunyamin Ece, Sonay Aydin

**Affiliations:** 1 Department of Ophthalmology, Kastamonu University, Kastamonu 37150, Turkey; zyozgat@kastamonu.edu.tr (Z.Y.); inancliyusuf@gmail.com (Y.İ.); 2 Department of Radiology, Kastamonu University, Kastamonu 37150, Turkey; bunyaminece@kastamonu.edu.tr; 3 Department of Radiology, Erzincan Binali Yıldırım University, Erzincan 24100, Turkey; sonay.aydin@erzincan.edu.tr

**Keywords:** penetrating globe injury, open globe injury, computed tomography, ocular trauma score, globe wall irregularity

## Abstract

**Background/Objectives**: The aim of this study was to evaluate computed tomography (CT) findings in penetrating globe injuries and their relationship with ocular trauma scores (OTSs). **Methods**: Patients with penetrating globe injuries who had orbital CT images were included in this study. Demographics, injury zone, and ophthalmologic exam data were collected retrospectively. OTSs and categories were determined. Orbital CT images were evaluated by a radiologist to determine decreased globe volume, globe wall irregularity, chorioretinal layer thickening, lens dislocation, asymmetric anterior chamber depth (ACD), abnormal vitreous density, and intraocular air presence. **Results**: This study included 30 eyes of 30 patients. The majority of patients (*n* = 20, 66.7%) had Zone I injuries. The most common CT findings were globe wall irregularity (53.3%) and asymmetric ACD (53.3%). No CT findings were observed in 10 patients (33.3%). Globe wall irregularity was the most frequent CT finding in the OTS II category, while asymmetric ACD was most frequent in the OTS IV category. All patients with decreased globe volume, lens dislocation, abnormal vitreous density, and ≥3 CT findings were in the OTS II category. A significant negative correlation was found between the number of CT findings and OTS (r = −0.644, *p* < 0.001), and a significant positive correlation was found between the number of CT findings and clinically measured wound size (r = 0.600, *p* < 0.001). **Conclusions**: CT findings help assess ocular trauma severity, but clinical examination remains essential for accurate diagnosis.

## 1. Introduction

Open globe injury (OGI) refers to a full-thickness wound of the eye wall (sclera and/or cornea) regardless of the involvement of the choroid or retina [1,2]. It is a significant cause of vision loss, especially in young working-age populations. Early diagnosis and prompt surgical intervention are critical for better outcomes. Eye injuries are classified as rupture and laceration according to the Birmingham Eye Trauma Terminology (BETT) system, and lacerations are further classified as penetrating, perforating, and intraocular foreign body based on the presence of entry and exit wounds. Penetrating globe injury is an OGI caused by a single entry, and if multiple injury sites are present, each must be caused by a different agent by definition [3].

Computed tomography (CT) is an effective tool in evaluating OGI, especially in patients with multiple trauma where ophthalmologic examination might be delayed or when ophthalmologic examination is difficult due to poor patient cooperation or periorbital soft tissue edema, providing valuable information about the integrity of the globe and intraocular structures [4,5]. CT findings are critical when deciding whether surgical exploration is necessary for suspected occult OGI. However, the sensitivity and specificity of CT in OGI patients are not optimal and vary across different studies. Yuan et al. reported the sensitivity and specificity of CT in OGI as 76% and 85%, respectively, while Joseph et al. reported 75% and 93%, respectively [6,7]. Nevertheless, as CT is the first diagnostic test performed to evaluate the extent and severity of acute injuries involving ocular trauma, the frequency and number of CT findings in OGI and their relationship with ophthalmologic examination findings can play an effective role in approaching the patient.

Previous studies have reported various CT findings in OGI patients, including alterations in globe contour, reduction in globe volume, changes in vitreous density, variations in anterior chamber depth, intraocular air, intraocular foreign body, and lens dislocation [6,7,8,9]. Although different CT findings have been reported in eyes with OGI, no study has specifically examined the CT findings of only penetrating eye injuries. This study aims to evaluate the type and frequency of CT findings in eyes with penetrating globe injury and to examine the relationship between CT findings and ophthalmologic examination characteristics and ocular trauma score (OTS).

## 2. Materials and Methods

**Figure 1:**
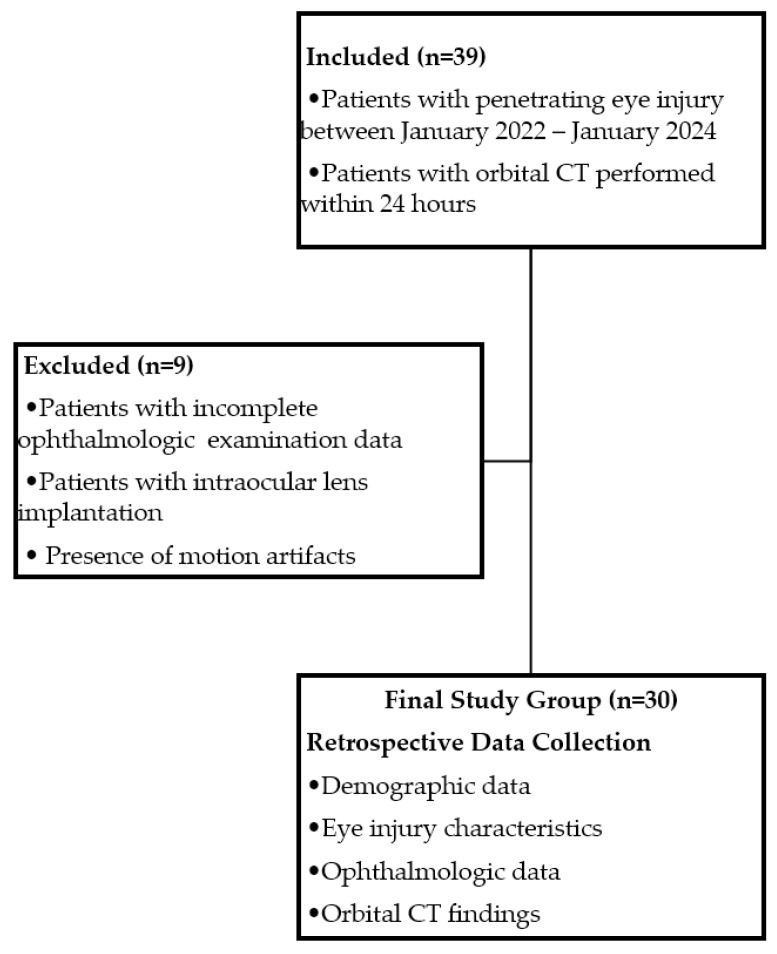
Flowchart of patient selection and data collection process.

This study included patients diagnosed with penetrating ocular injury via ophthalmological examination between January 2022 and January 2024 who had available orbital CT images obtained within 24 h. Ethical approval was obtained for this study (2024-KAEK-31). Due to the retrospective design of this study, additional informed consent was not required outside of the routine general information and consent form obtained before imaging methods and the consent form obtained before the repair of open globe injuries. This study adhered to the principles of the Helsinki Declaration. Patients with rupture, perforating injury, or intraocular foreign body, those with incomplete data in ophthalmologic examination records, those with previous intraocular lens implantation, and those with orbit CT images unsuitable for evaluation due to motion artifacts were excluded from this study (See Figure 1 for the study design flowchart).

Demographic data of the patients, the mechanism of injury, location of globe injury, best corrected visual acuity (BCVA), hyphema, vitreous hemorrhage, relative afferent pupillary defect (RAPD), presence of orbital fracture, and retinal detachment were retrospectively collected from patient files. BCVA was determined using the Snellen scale and converted to the logarithm of the minimum angle of resolution (LogMAR) for statistical analysis. Extremely low visual acuity values were considered as follows: counting fingers 1.9 LogMAR, hand movements 2.3 LogMAR, perception of light 2.7 LogMAR, and no light perception 3.0 LogMAR [10,11]. Patients were classified into three anatomical zones according to the injury location: Zone I (cornea and corneoscleral limbus), Zone II (up to 5 mm behind the corneoscleral limbus), and Zone III (posterior to Zone II, including the macula and optic nerve) [12]. The OTS of the patients was calculated as a numerical value between 0 and 100 according to the method proposed by Kuhn et al., assigning a raw score value to initial visual acuity and subtracting points based on the presence of endophthalmitis, globe rupture or perforating injury (these patients were not included in our study), retinal detachment, and RAPD. Patients were then divided into five OTS categories based on this numerical value [13]. A higher OTS is associated with a better predicted prognosis, while a lower OTS is linked to a worse predicted prognosis [14].

Orbital CT images of patients were retrospectively evaluated for decreased globe volume, irregularity of the globe wall, thickening of the chorioretinal layer, lens dislocation, asymmetric anterior chamber depth (ACD), abnormal vitreous density, and presence of intraocular air by a radiologist that was blinded to clinical features. Asymmetric ACD was defined as an ACD difference of 0.4 mm or more (increase or decrease) between the affected and contralateral eyes. ACD measurements were made on the axial plane, at the level of the globe equator, with a vertical line drawn from the back of the cornea to the front surface of the lens. Patients with lens dislocation were excluded from the measurement. In patients with detectable irregularities in the globe wall where globe volume loss did not impede measurement, the distance of the irregularity was measured. The type and frequency of CT findings were recorded to assess their relationship with ophthalmological examination findings and OTS.

CT imaging was performed using a 64-detector 128-slice multi-detector CT scanner (Revolution EVO, GE Medical System, Chicago, IL, USA). Imaging parameters were as follows: 120 kV, 100–350 mA, and 0.5 to 0.625 s portal rotation time. The slice thickness of the axial CT images was 0.625 mm, while the coronal and sagittal reformatted images had a slice thickness of 1.25 mm. All imaging was performed without contrast. Evaluations were performed by a radiologist with 11 years of experience using an Advantage Windows workstation (ADW 4.7 Ext. 16 Software, GE Medical System, Chicago, IL, USA).

### Statistical Analysis and Power Assessment

Descriptive statistics of the data were expressed as mean, standard deviation, frequency, and percentage values. The distribution of quantitative variables was measured using the Shapiro–Wilk tests. Spearman correlation analysis was used to evaluate the correlation between the number of CT findings, OTS, and wound sizes (measured clinically and via CT). *p*-values less than 0.05 were considered statistically significant. Statistical analysis was performed using SPSS software, version 26.0 (IBM SPSS, Chicago, IL, USA).

A post hoc power analysis was conducted to assess the statistical robustness of our findings using G*Power software (version 3.1.9.7). For all correlation analyses, we employed the “Exact” test family with “Correlation: Bivariate normal model” as the statistical test. Power calculations were performed using a two-tailed significance level (α) of 0.05 and our sample size of 30 patients.

## 3. Results

The study included 30 eyes of 30 patients with penetrating eye injury. The mean age of the patients was 49.4 ± 20.0 years (range 4–74). Of the patients, 26 (86.7%) were male. Injuries were in the right eye in 13 patients (43.3%) and the left eye in 17 patients (56.7%). Metal objects were the most common cause of injury (*n* = 13, 43.3%). The mean visual acuity was 2.05 ± 0.74 LogMAR, and the mean wound size was 4.7 ± 2.7 mm according to clinical examination. The majority of injuries (*n* = 20, 66.7%) were in Zone I. The OTS categories of the patients are shown in Table 1. In this study, no patients were in OTS categories I and V.

**Figure 2:**
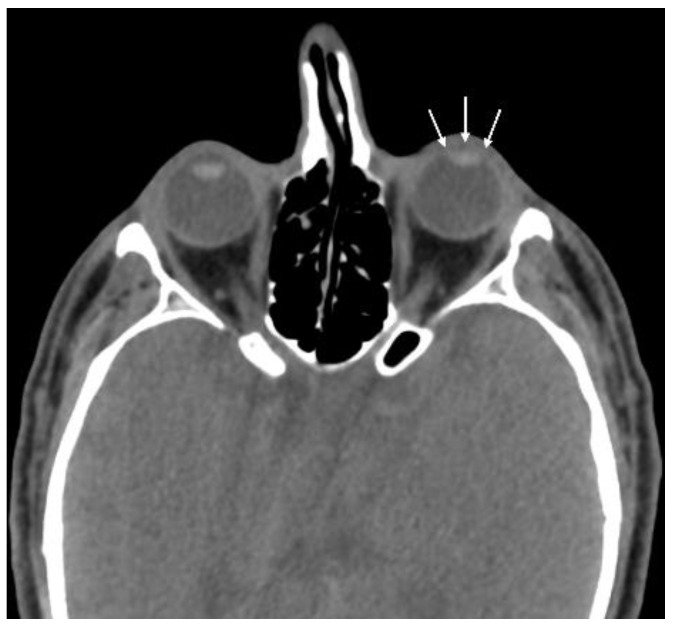
Patient with penetrating globe injury in the left eye. The patient exhibits asymmetric anterior chamber depth (arrows) and is classified in ocular trauma score category IV.

**Figure 3:**
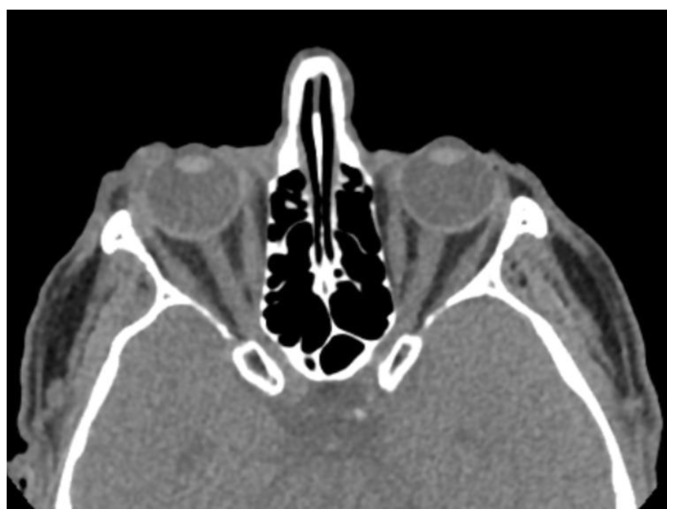
Patient with penetrating globe injury in the right eye. The patient has no CT findings and is classified in ocular trauma score category III.

The most common CT findings were globe wall irregularity (53.3%) and asymmetric ACD (53.3%) (Figure 2). No CT findings were detected in 10 patients (33.3%) (Figure 3). The sensitivity of CT in patients with penetrating eye injury was determined to be 66.7%. The type and number of CT findings in the patients are shown in Table 2. The mean distance of globe wall irregularity measured by CT evaluation was 2.2 ± 0.9 mm. A significant positive correlation was found between the clinically detected wound size and the distance of globe wall irregularity detected by CT (r = 0.656, *p* < 0.05).

**Figure 4:**
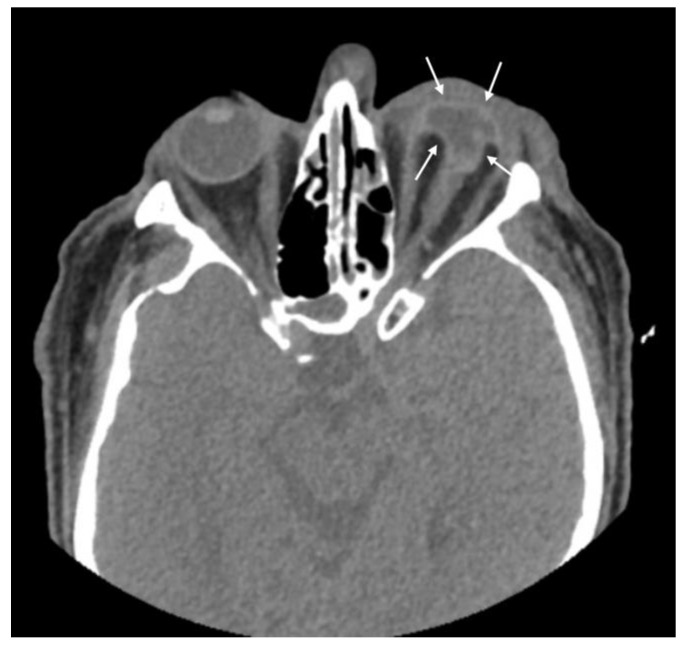
Patient with penetrating globe injury in the left eye. The patient exhibits globe volume loss (arrows) and is classified in ocular trauma score category II.

**Figure 5:**
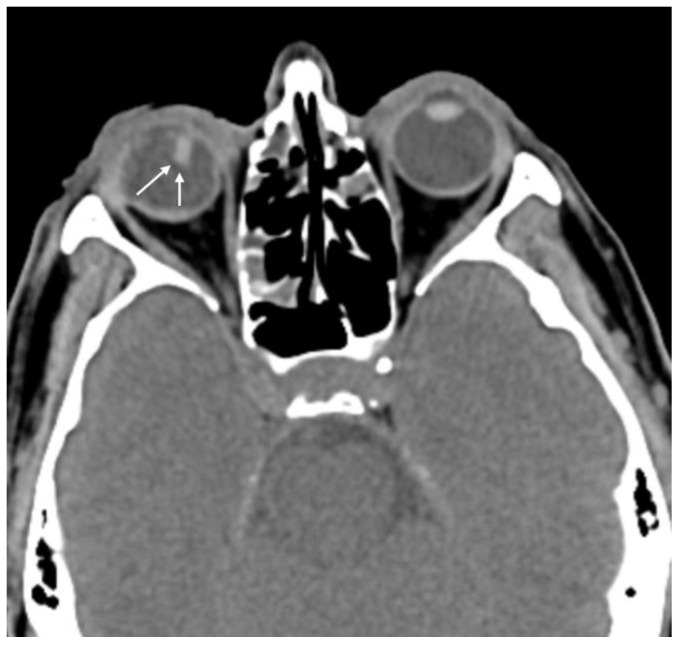
Patient with penetrating globe injury in the right eye. The patient exhibits lens dislocation (arrows) and is classified in ocular trauma score category II.

The distribution of CT findings by OTS category (II, III, and IV) is shown in Table 3. Accordingly, the most common category CT finding in OTS II was globe wall irregularity, while the most common finding in category IV was asymmetric ACD. Patients with decreased globe volume (Figure 4), lens dislocation (Figure 5), and abnormal vitreous density were all in OTS category II.

**Figure 6:**
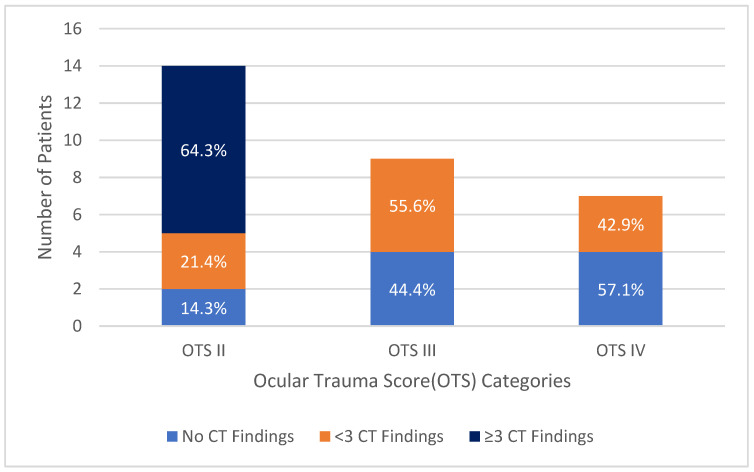
Number of computed tomography findings by ocular trauma score categories.

The distribution of the number of CT findings by OTS category is shown in Figure 6. All patients with three or more CT findings were in OTS category II. A significant negative correlation was found between the number of CT findings and OTS (r = −0.644, *p* < 0.001). The mean OTS of patients with no CT findings was 80.0 ± 11.5, the mean OTS of patients with fewer than three CT findings was 72.7 ± 11.9, and the mean OTS of patients with three or more CT findings was 59.7 ± 5.0. A significant positive correlation was found between the number of CT findings and the clinically detected wound size (r = 0.600, *p* < 0.001).

A post hoc power analysis was performed to assess the robustness of the key correlations in our study, and the results demonstrated strong statistical validity. The correlation analysis between the number of CT findings and ocular trauma score (OTS) demonstrated robust statistical reliability, with a calculated post hoc power of 97.8%. Similarly, the relationship between clinically detected wound size and CT-detected globe wall irregularity distance exhibited strong statistical validity, with a post hoc power of 98.3%. Furthermore, the correlation between the number of CT findings and clinically detected wound size yielded a post hoc power of 95.0%.

## 4. Discussion

In our study, we included only patients with penetrating globe injuries and focused on the relationship between CT findings and OTS. In perforating injuries with different developmental mechanisms, the presence of both entry and exit points of the foreign body or the permanence of the foreign body in the presence of an intraocular foreign body may potentially cause different CT findings and different numbers of CT findings. Due to our inclusion of only penetrating globe injuries in our study, our results are important in terms of drawing attention to the presence of conditions such as asymmetric anterior chamber or absence of any CT findings that might be overlooked if not carefully examined, as in corneal wound.

In our study, the most common CT findings in patients with penetrating eye injury were globe wall irregularity (53.3%) and asymmetric ACD (53.3%). Hoffstetter et al. reported globe deformation as the most common CT finding in open globe injuries in their study [15]. Ameli et al. reported the most common CT findings in OGI patients as scleral irregularity or globe collapse (71.9%) and abnormal vitreous density (56%) [16]. Arabi et al. reported the most common CT findings as scleral irregularity (70%), lens dislocation (54%), vitreous hemorrhage (51%), and asymmetric ACD (47%) [9]. The higher incidence of asymmetric ACD in our study compared to similar studies might be due to the inclusion of only penetrating injuries and due to the majority of injuries being in Zone I. In another study examining CT findings of open globe injuries in the pediatric population, the most common CT findings were reported as scleral irregularity and increased preseptal thickness (47.1%) [17]. The current findings indicate that scleral wall irregularity is the most common finding in globe injuries; therefore, if detected, it is important to also review other possible signs of open globe injury.

In our study, we considered both an increase and a decrease in the ACD of the affected eye compared to the contralateral eye as positive CT findings. This is because anterior corneoscleral lacerations can cause a flattening of the anterior chamber, while posterior scleral lacerations can result in lens retropulsion and an increase in ACD due to reduced vitreous pressure. Weissman et al. reported three patients with posterior globe rupture in whom they observed an increase in ACD. They noted that a difference of 2 mm or more in ACD measurements between the two eyes on CT increases the likelihood of a scleral rupture [18]. In another study, the mean ACD in eyes with OGI was reported to be 1.5 mm, while it was 3.1 mm in the contralateral eyes [4]. Moreover, Kim et al. reported that an ACD measurement difference of ≥ 0.4 mm between the two eyes had a sensitivity of 73% and specificity of 100% in detecting OGI on CT [19]. In addition, Parlak et al. found significantly lower ACD in OGI patients and reported the best cut-off value for ACD in detecting globe injury as 2.475 mm with 73.7% sensitivity and 88% specificity [20]. In the literature, there are studies recommending the quantitative assessment of ACD and density, rather than solely examining volume loss or globe deformation, to avoid missing occult injuries [21]. Therefore, measuring and comparing ACD with the contralateral eye can provide clinical benefit by detecting positive CT findings in suspected cases.

Despite the presence of penetrating globe injury, CT findings were absent in 33.3% of patients. In previous studies, the presence of normal CT images in eyes with OGI has been reported to range from 10% to 55% [22,23,24,25]. The absence of CT findings in one-third of our patients may not be due to the technical limitations of CT but rather to the fact that the type of OGI included in our study was penetrating trauma and that the majority of injuries were located in Zone I. Additionally, we determined the sensitivity of CT in penetrating eye injury to be 66.7%. In previous studies, the sensitivity of CT in detecting OGI in the absence of clinical information was reported as 70–75% [6,7,15]. Arabi et al. [9] reported the positive predictive value of CT in OGI as 91% and the negative predictive value as 84%, while Arey et al. [8] reported the positive predictive value as 89% and the negative predictive value as 52%. Our results, along with the relatively low negative predictive values reported in different studies, suggest that trying to detect penetrating globe injury solely with CT without clinical examination may not provide sufficient accuracy.

In approximately 10% of our patients, intraocular air was detected on CT. Although less frequently detected compared to other CT findings, the presence of intraocular air is significant because it is suggested to be a pathognomonic sign of OGI if air is found inside the globe on CT after trauma. Arey et al. reported that one of six globes with intraocular air detected on CT was found to be intact during surgical exploration, but later, during pars plana vitrectomy, findings suggestive of a small penetrating injury that had been missed during the initial examination were noted [8].

Penetrating globe injuries present challenges such as providing patient counseling and informing about visual prognosis. Studies in the literature have shown that OTS supports ophthalmologists in determining surgical timing after OGI, assessing the need for early vitreoretinal surgery, defining the surgical scope, managing secondary complications and secondary surgical requirements, and predicting visual acuity outcomes [26,27,28,29,30,31]. In our study, the negative correlation between the number of CT findings and OTS, as well as the presence of certain CT findings (decreased globe volume, lens dislocation, and abnormal vitreous density) and the fact that all patients with ≥3 CT findings were classified in OTS category II, suggest that the evaluation of CT findings may also play a role in this critical process. Similarly, Ameli et al. reported that abnormal vitreous density, chorioretinal thickening, and the presence of intraocular foreign body/air were associated with more advanced OTS stages (I-II). They also reported lower OTS and more advanced OTS categories in eyes with more CT findings [16]. The association of certain CT-detected findings with more advanced OTS categories in our study and similar studies may be related to the fact that these pathologies contribute to lower initial visual acuity. Arey et al. found significantly higher positive radiographic findings on CT in eyes with occult OGI compared to intact eyes [8]. Additionally, another study reported that patients with poor visual outcomes (<2/200) or those who underwent enucleation demonstrated significantly more CT abnormalities compared to patients with good visual outcomes [7]. Our results and those in the literature suggest that detecting multiple positive CT findings in trauma patients with OGI should be noteworthy to ophthalmologists. Particularly in cases where initial visual assessment cannot be performed due to conditions such as loss of consciousness, CT may help in managing post-traumatic uncertainty.

A previous study reported that while there was no significant difference between axial and coronal CT scans in detecting OGI, no CT evaluator was able to comment on ACD using coronal sections. Consequently, they highlighted that coronal CT examination is not sufficiently sensitive for detecting posterior globe ruptures. The same study reported the sensitivity and specificity of CT in detecting OGI as 74% and 90% for axial sections and 65% and 89% for coronal sections, respectively, with sensitivity and specificity increasing to 79% and 93% with combined examination [9]. Therefore, in our study, we aimed to identify the maximum number of positive findings related to penetrating globe injuries by using a combination of axial, coronal, and sagittal images during patient evaluation.

Our study has some limitations. The retrospective design and relatively small sample size are among these limitations. The inclusion of only one subtype of OGI and the exclusion of pseudophakic patients to better evaluate ACD asymmetry contributed to the small sample size. However, our post hoc power analysis yielded results ranging from 95.0% to 98.3%. These power values substantially exceed the conventionally accepted threshold of 80%, indicating that our sample size was sufficient to detect the observed effects with high statistical confidence. Furthermore, the strength of the observed correlations ensured robust statistical power, minimizing the probability of Type II errors. Additionally, the inclusion of patients with only three OTS categories further limits this study. Another significant limitation is the lack of inter- and intra-observer agreement analysis due to the CT findings being evaluated by a single radiologist in one session. Additionally, although CT images were evaluated by a radiologist blinded to clinical information, structured finding lists may have increased sensitivity in detecting CT findings. Despite these limitations, to our knowledge, our study is the first to examine CT findings in patients with penetrating globe injury only.

## 5. Conclusions

In conclusion, the type and number of CT findings in patients with penetrating eye injury provide useful information in determining the severity of ocular trauma. However, detecting penetrating eye injuries solely based on CT evaluation without clinical assessment is insufficient.

## Figures and Tables

**Table 1:** Basic clinical characteristics of patients with penetrating globe injury.

Clinical Characteristics (*n* = 30)	
Age (years)	49.4 ± 20.0
Gender (F/M), *n* (%)	26 (86.7)/4 (13.3)
Injury Zone, *n* (%)	
Zone I	20 (66.7)
Zone II	7 (23.3)
Zone III	3 (10.0)
Object of Injury, *n* (%)	
Metal	13 (43.3)
Glass	8 (26.7)
Wood	7 (23.3)
Horn	2 (6.7)
Visual Acuity (logMAR) (Mean ± SD)	2.05 ± 0.74
Wound Size (mm) (Mean ± SD)	4.7 ± 2.7
OTS Category	
I (0–44)	0
II (45–65)	14 (46.7)
III (66–80)	9 (30.0)
IV (81–91)	7 (23.3)
V (92–100)	0

logMAR: logarithm of the minimum angle of resolution; OTS: ocular trauma score; Mean ± SD: Mean ± standard deviation.

**Table 2:** Computed tomography findings in patients with penetrating globe injury.

CT Findings	*n* (%)
**Type of CT Findings**	
Globe wall irregularity	16 (53.3)
Asymmetric anterior chamber depth	16 (53.3)
Decreased globe volume	6 (20.0)
Chorioretinal layer thickening	5 (16.7)
Abnormal vitreous density	5 (16.7)
Lens dislocation	3 (10.0)
Intraocular air	3 (10.0)
**Number of CT Findings**	
None	10 (33.3)
1 CT finding	2 (6.7)
2 CT findings	9 (30.0)
3 CT findings	5 (16.7)
4 CT findings	2 (6.7)
≥ 5 CT findings	2 (6.7)

CT: computed tomography.

**Table 3:** Computed tomography findings by ocular trauma score category.

OTS Category	Globe Wall Irregularity	Asymmetric ACD	Decreased Globe Volume	Chorioretinal Thickening	Abnormal Vitreous Density	Lens Dislocation	Intraocular Air
OTS II (*n* = 14)	10 (71.4)	9 (64.3)	6 (42.9)	4 (28.6)	5 (35.7)	3 (21.4)	2 (14.3)
OTS III (*n* = 9)	4 (44.4)	4 (44.4)	0	0	0	0	1 (11.1)
OTS IV (*n* = 7)	2 (28.6)	3 (42.9)	0	1 (14.3)	0	0	0

OTS: ocular trauma score; ACD: anterior chamber depth.

## Data Availability

The data presented in this study are available on request from the corresponding author. The data are not publicly available due to ethical restrictions.

## Abbreviations

The following abbreviations are used in this manuscript:

CT	Computed tomography
OTS	Ocular trauma score
ACD	Anterior chamber depth
OGI	Open globe injury
BETT	Birmingham Eye Trauma Terminology
BCVA	Best corrected visual acuity
RAPD	Relative afferent pupillary defect
LogMAR	Logarithm of the minimum angle of resolution
